# Research Progress on the use of Plant Allelopathy in Agriculture and the Physiological and Ecological Mechanisms of Allelopathy

**DOI:** 10.3389/fpls.2015.01020

**Published:** 2015-11-17

**Authors:** Fang Cheng, Zhihui Cheng

**Affiliations:** College of Horticulture, Northwest A&F University, Yangling, China

**Keywords:** allelochemical, allelopathy, agriculture practice, physiological mechanism, ecological mechanism, microorganism, agricultural sustainable development

## Abstract

Allelopathy is a common biological phenomenon by which one organism produces biochemicals that influence the growth, survival, development, and reproduction of other organisms. These biochemicals are known as allelochemicals and have beneficial or detrimental effects on target organisms. Plant allelopathy is one of the modes of interaction between receptor and donor plants and may exert either positive effects (e.g., for agricultural management, such as weed control, crop protection, or crop re-establishment) or negative effects (e.g., autotoxicity, soil sickness, or biological invasion). To ensure sustainable agricultural development, it is important to exploit cultivation systems that take advantage of the stimulatory/inhibitory influence of allelopathic plants to regulate plant growth and development and to avoid allelopathic autotoxicity. Allelochemicals can potentially be used as growth regulators, herbicides, insecticides, and antimicrobial crop protection products. Here, we reviewed the plant allelopathy management practices applied in agriculture and the underlying allelopathic mechanisms described in the literature. The major points addressed are as follows: (1) Description of management practices related to allelopathy and allelochemicals in agriculture. (2) Discussion of the progress regarding the mode of action of allelochemicals and the physiological mechanisms of allelopathy, consisting of the influence on cell micro- and ultra-structure, cell division and elongation, membrane permeability, oxidative and antioxidant systems, growth regulation systems, respiration, enzyme synthesis and metabolism, photosynthesis, mineral ion uptake, protein and nucleic acid synthesis. (3) Evaluation of the effect of ecological mechanisms exerted by allelopathy on microorganisms and the ecological environment. (4) Discussion of existing problems and proposal for future research directions in this field to provide a useful reference for future studies on plant allelopathy.

## Introduction

Allelopathy is a sub-discipline of chemical ecology that is concerned with the effects of chemicals produced by plants or microorganisms on the growth, development and distribution of other plants and microorganisms in natural communities or agricultural systems (Einhellig, 1995). The study of allelopathy increased in the 1970s and has undergone rapid development since the mid-1990s, becoming a popular topic in botany, ecology, agronomy, soil science, horticulture, and other areas of inquiry in recent years. The allelopathic interaction can be one of the significant factors contributing to species distribution and abundance within plant communities and can be important in the success of invasive plants (Chou, 1999; Mallik, 2003; Field et al., 2006; Inderjit et al., 2006; Zheng et al., 2015), such as water hyacinth (*Eichhornia crassipes* Mart. Solms) (Jin et al., 2003; Gao and Li, 2004), spotted knapweed (*Centaurea stoebe* L. ssp. *micranthos*) (Broeckling and Vivanco, 2008) and garlic mustard (*Alliaria petiolata* M. Bieb) (Vaughn and Berhow, 1999). Allelopathy is also thought to be one of the indirect causes of continuous cropping obstacles in agriculture. As a result of the in-depth study of allelopathy, strategies for the management of agricultural production and ecological restoration involving the application of allelopathy and allelochemicals are improving. The main purposes of this review are to present conclusions regarding the application of allelopathy in agricultural production, to highlight the physiological and ecological mechanisms underlying plant allelopathy, to illustrate the effect of allelopathy on soil microorganisms and to discuss key points for further research.

## Allelopathy and Allelochemicals

The definition of allelopathy was first used by Molish in 1937 to indicate all of the effects that directly and indirectly result from biochemical substances transferred from one plant to another (Molisch, 1937). Almost half a century later, the accepted targets of allelochemicals in the plant kingdom include algae, fungi and various microorganisms. The term was refined by Rice (1984) to define “any direct or indirect harmful or beneficial effect by one plant (including microorganisms) on another through production of chemical compounds that escape into the environment” (Rice, 1984). In 1996, the International Allelopathy Society broadened its definition of allelopathy to refer to any process involving secondary metabolites produced by plants, microorganisms, viruses and fungi that influence the growth and development of agricultural and biological systems. In addition, the allelopathic donor and receiver should include animals (Kong and Hu, 2001).

**FIGURE 1:**
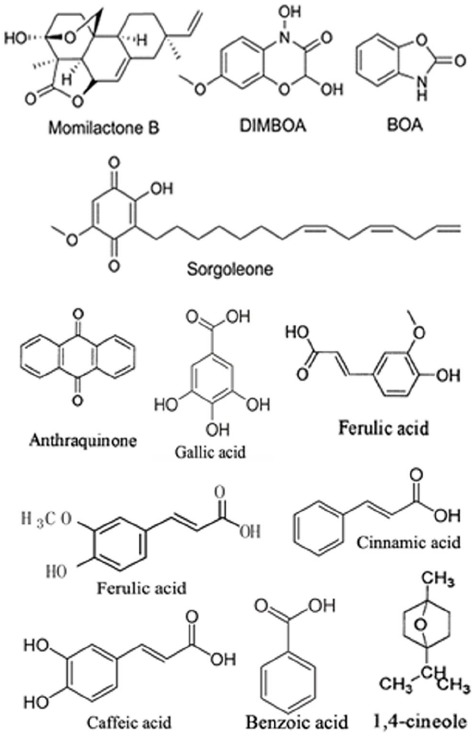
**Structures of some of the allelochemicals produced by plants**.

Allelochemicals, which are non-nutritive substances mainly produced as plant secondary metabolites or decomposition products of microbes, are the active media of allelopathy. Allelochemicals consist of various chemical families and are classified into the following 14 categories based on chemical similarity (Rice, 1974): water-soluble organic acids, straight-chain alcohols, aliphatic aldehydes, and ketones; simple unsaturated lactones; long-chain fatty acids and polyacetylenes; benzoquinone, anthraquinone and complex quinones; simple phenols, benzoic acid and its derivatives; cinnamic acid and its derivatives; coumarin; flavonoids; tannins; terpenoids and steroids; amino acids and peptides; alkaloids and cyanohydrins; sulfide and glucosinolates; and purines and nucleosides. Plant growth regulators, including salicylic acid, gibberellic acid and ethylene, are also considered to be allelochemicals. The rapid progress of analysis technology in recent years has made it possible to isolate and identify even minute amounts of allelochemicals and to perform sophisticated structural analyses of these molecules. The structures of some allelochemicals produced by plants are shown in Figure 1.

## Management of Plant Allelopathy in Agriculture

Allelopathy is a natural ecological phenomenon. It has been known and used in agriculture since ancient times (Zeng, 2008, 2014). Allelochemicals can stimulate or inhibit plant germination and growth, and permit the development of crops with low phytotoxic residue amounts in water and soil, thus facilitating wastewater treatment and recycling (Macias et al., 2003; Zeng et al., 2008). They are a suitable substitute for synthetic herbicides because allelochemicals do not have residual or toxic effects, although the efficacy and specificity of many allelochemicals are limited (Bhadoria, 2011). The main purposes of research on allelopathy include the application of the observed allelopathic effects to agricultural production, reduction of the input of chemical pesticides and consequent environmental pollution, and provision of effective methods for the sustainable development of agricultural production and ecological systems (Macias et al., 2003; Li et al., 2010; Han et al., 2013; Jabran et al., 2015). The use of allelopathic crops in agriculture is currently being realized, e.g., as components of crop rotations, for intercropping, as cover crops or as green manure (Cheema and Khaliq, 2000; Singh et al., 2003; Cheema et al., 2004; Khanh et al., 2005; Reeves et al., 2005; Yildirim and Guvenc, 2005; Iqbal et al., 2007; Mahmood et al., 2013; Wortman et al., 2013; Farooq et al., 2014; Silva et al., 2014; Wezel et al., 2014; Haider et al., 2015). The applications of allelopathy in crop production in Pakistan are successful examples in recent years (Cheema et al., 2013). The suitable application of allelopathy toward the improvement of crop productivity and environmental protection through environmentally friendly control of weeds, insect pests, crop diseases, conservation of nitrogen in crop lands, and the synthesis of novel agrochemicals based on allelochemicals has attracted much attention from scientists engaged in allelopathic research.

## Arrangement of Cropping Systems

Competition is one of the main modes of interaction between cultivated crops and their neighboring plants (Inderjit and Moral, 1997; Xiong et al., 2005; He et al., 2012b; An et al., 2013). Allelopathy is a chemical mechanism that provides plants with an advantage for competing for limited resources (Singh et al., 1999; He et al., 2012b; Gioria and Osborne, 2014). The ability of plants to suppress weeds is thus determined by crop allelopathy and competitiveness. Crop allelopathy can be effectively used to control weeds in the field, to alleviate allelopathic autotoxicity and reduce inhibitory influence among allelopathic crops (Iqbal et al., 2007; John et al., 2010; Farooq et al., 2013; Andrew et al., 2015), to improve the utilization rate of land and to increase the annual output of the soil by establishing reasonable crop rotation and intercropping systems. For example, Odeyemi et al. (2013) reported relative abundance and population suppression of plant parasitic nematodes under *Chromolaena odorata* (L.) (Asteraceae) fallow in a field study conducted over 2 years, and suggested that the use of bush fallow with *C. odorata* might become an integrated management practice in the management of nematode pests in crop production in south-western Nigeria. Intercropping is a common practice among farmers in developing countries for maximizing land resources and reducing the risks of single crop failure. Weed population density and biomass production can be markedly reduced using crop rotation and intercropping systems (Liebman and Dyck, 1993; Narwal, 2000; Nawaz et al., 2014; Jabran et al., 2015). Intercropping of sorghum (*Sorghum bicolor* L.), sesame (*Sesamum indicum* L.) and soybean (*Glycine max* L.) in a cotton (*Gossypium hirsutum* L.) field produced greater net benefits and a significant inhibition on purple nutsedge (*Cyperus rotundus* L.) in comparison with the cotton alone in a 2-year experiment (Iqbal et al., 2007). Recently, Wang et al. (2015) reported that eggplant/garlic relay intercropping is a beneficial cultivation practice to maintain stronger eggplant growth and higher yield. However, the allelopathy between different species may cause promontory or inhibitory effects. Farooq et al. (2014) reported that when grown in rotation with tobacco (*Nicotiana tabacum* L.), the stand establishment and growth of maize (*Zea mays* L.) were improved compared to mung bean (*Vigna radiata* L.), whereas mungbean stand establishment and growth were suppressed. Therefore, the allelopathic nature of crops must be considered in crop rotation, intercropping and stalk mulching (Xuan et al., 2005; Cheng et al., 2011; Cheng and Xu, 2013).

## Straw Mulching

**FIGURE 2:**
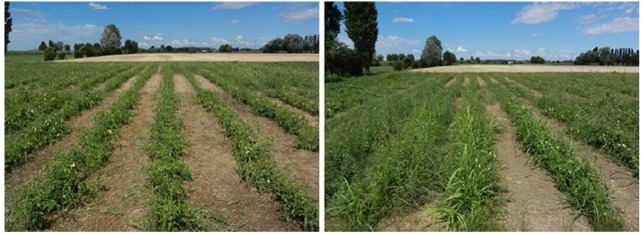
**Field trial on rye mulch preceding a tomato crop in a biological farm (**Schulz et al., 2013**).** Left, test plot with rye mulch left on the soil surface, showing the good weed suppression ability. Right, control plot without rye mulch, split into two treatments: left side, untreated sub-plot in which tomato plants are almost completely overgrown by weeds; right side, sub-plot with mechanical control by cultivation, in which tomato plants grow as well as those in the test plot.

In conventional agriculture, weed control using herbicides is not only an expensive practice; it is also harmful to the environment. Allelopathic applications, such as straw mulching, provide sustainable weed management (Jabran et al., 2015), further reducing the negative impact of agriculture on the environment (Cheema and Khaliq, 2000; Cheema et al., 2004). Using allelopathic plants as ground cover species provides an environmental friendly option (Dhima et al., 2006; Moraes et al., 2009; Wang et al., 2013a). The allelochemicals from decomposed straw can suppress weed growth in farmlands, and reduce the incidence of pests and diseases. Moreover, straw mulch can improve the soil organic matter content and increase soil fertility. However, it may also have negative effects by increasing the C: N ratio of the soil. Research has shown that green wheat (*Triticum aestivum* L.) straw inhibits the growth of *Ipomoea* weeds in corn (*Zea mays* L.) and soybean fields, thereby reducing the need for herbicide application. Rye (*Secale cereale* L.) mulch significantly reduced the germination and growth of several problematic agronomic grass and broadleaf weeds (Figure 2; Schulz et al., 2013). The transformation reactions of rye allelochemicals, i.e., benzoxazinoids, in soil led primarily to the production of phenoxazinones, which can be degraded by several specialized fungi via the Fenton reaction. Because of their selectivity, specific activity, and presumably limited persistence in the soil, benzoxazinoids or rye residues are suitable means for weed control (Schulz et al., 2013). Furthermore, Tabaglio et al. (2008) found that the allelopathic inhibition effects on weeds differ between different cultivars of rye straw used for mulching. Xuan et al. (2005) concluded that the application of allelopathic plant materials at 1–2 tons ha^–1^ could reduce weed biomass by approximately 70%, and increase rice (*Oryza sativa* L.) yield by approximately 20% in paddy fields (1998–2003) compared with the respective controls. In the southeastern region of Brazil, coffee (*Coffea arabica*) fruit peels, which contain allelochemicals such as phenols, flavonoids and caffeine, are often used as an organic amendment in agricultural practice to control weeds (Silva et al., 2013). An et al. (2013) found that switchgrass (*Panicum virgatum* L.) plants and residues reduced the biomass and density of associated weeds, and their research provided weed management strategies in agroecosystems and important information for the introduction of switchgrass into new ecosystems. Water extracts of *Conyza bonariensis* (L.) Cronquist, *Trianthema portulacastrum* L., and *Pulicaria undulata* (L.) C. A. Mey. can be applied at a concentration of 10 g L^–1^ to manage the weed *Brassica tournefortii* Gouan by inhibiting germination and seedling growth (Abd El-Gawad, 2014). Moreover, some soybeans induce the germination of sunflower broomrape (*Orobanche* spp.), a noxious parasitic weed, which suggests that soybean has the potential to be used as a trap crop to reduce the seed bank of sunflower broomrape (Zhang et al., 2013b).

## Developing Environmentally Friendly Agrochemical and Microbial Pesticides

Allelochemicals with negative allelopathic effects are important components of plant defense mechanisms against weeds and herbivory. The technology that modifies allelochemicals for the production of environmentally friendly pesticides and plant growth regulators allows the effective management of agricultural production and confers few environmental problems in the soil due to the fairly high degradability of allelochemicals (Bhadoria, 2011; Ihsan et al., 2015). Uddin et al. (2014) revealed that sorgoleone, a hydrophobic compound found in *Sorghum bicolor* (L.) root exudates, was more effective in inhibiting weed growth after formulation as a wettable powder, while crop species were tolerant to it. Some microorganisms are capable of using sorgoleone as a carbon source. Sorgoleone can be mineralized via complete degradation to CO_2_ in soil, although the different chemical groups of the molecule were not mineralized equally (Gimsing et al., 2009). The strong weed-suppressive ability of formulated sorgoleone raised interest as an effective, natural, environmentally friendly approach for weed management. Plant growth-promoting rhizobacteria (PGPR) include a wide range of beneficial bacteria that confer positive effects on plants, such as eliciting induced systemic resistance (ISR), promoting plant growth and reducing susceptibility to diseases caused by plant pathogens (Kloepper et al., 1980, 2004). Allelopathic bacteria can achieve the same function in mixtures of bacteria that exhibit PGPR attributes and activity against allelopathic weeds, which reduces the inhibitory effect on susceptible plants caused by allelopathic weeds (Kremer, 2006; Mishra and Nautiyal, 2012). There are some organic herbicides or plant growth inhibitors that have been manufactured from allelopathic plant materials to inhibit weed growth in fields (Guillon, 2003; Ogata et al., 2008; Miyake, 2009). Ogata et al. (2008) manufactured a type of herbicide comprised of a mixture of components extracted from pine (*Pinus* L.), hinoki (*Chamaecyparis obtusa* Endl.), or Japanese cedar (*Cryptomeria japonica* D. Don) and bamboo (Bambusoideae; Poaceae) vinegar, which provided a practical method of utilizing plant allelopathy in paddy fields.

## Reduction of Nitrogen Leaching and Environmental Pollution

Nitrogen leaching is a severe ecological problem due to water pollution. Mineralization of soil organic nitrogen, especially the nitrification of nitrogen fertilizer, is one of the main reasons for the enrichment of nitrogen in the soil. Biological nitrification inhibition (BNI) has gradually become the main target in investigating the effect of plants on soil nitrification. In recent years, studies have proven that nitrification-inhibiting substances (NIS) produced by plants are the first choice for soil nitrification management. For example, biological nitrification inhibition substances (BNIS) are allelochemicals that are able to inhibit soil nitrification. Wheat allelochemicals, such as ferulic acid, p-hydroxybenzoic acid and hydroxamic acid, can act on soil microbes to inhibit soil nitrification, reduce the emission of N_2_O, improve the utilization rate of nitrogen fertilizer and reduce pollution to the environment (Ma, 2005). Dietz et al. (2013) found that the allelopathic plantain (*Plantago lanceolata* L.) plant has inhibitory effects on soil nitrogen mineralization, suggesting that plantain could be utilized to reduce soil nitrogen leaching.

## Breeding of Allelopathic Cultivars

Allelopathic cultivars, which have great potential to minimize the introduction of refractory chemicals and effectively control weeds in farmland ecosystems, represent the most promising application of allelopathy (Mahmoud and Croteau, 2002; Weston and Duke, 2003; Fragasso et al., 2013). Both conventional breeding methods and those developed using transgenic technology can be applied in the breeding of allelopathic cultivars. Successful cultivars must also combine a weed suppression ability with high yield potential, disease resistance, early maturity and quality traits (Gealy and Yan, 2012). Rondo, a rice cultivar that combines a high yield potential with rice blast resistance and weed suppression ability, has been grown in a commercial organic rice production operation in Texas and its weed-suppressive ability is superior to that of many commercial cultivars (Yan and McClung, 2010; Gealy and Yan, 2012). Huagan 3, a particularly promising F_8_ generation line derived from crosses between the local rice cultivars N9S and PI 312777, is considered to be the first commercially acceptable weed-suppressive cultivar in China (Kong et al., 2011). Bertholdsson (2010) bred spring wheat for improved allelopathic potential by conventional breeding. The material used originated from a cross between a Swedish cultivar with low allelopathic activity and a Tunisian cultivar with high allelopathic activity. The result from the field study was a 19% average reduction in weed biomass for the high allelopathic lines. However, a negative effect was that the grain yield was reduced by 9% in the high allelopathic lines. In this research, the high allelopathic lines showed a lower early biomass compared with the control. If the early biomass of the allelopathic wheat had also been improved, the weed biomass should have been much lower (Bertholdsson, 2004). Putative genes related to the weed competition ability of wheat have been found on chromosomes 1A, 2B, and 5D via quantitative trait locus (QTL) identification, which might be helpful for the breeding of allelopathic wheat (Zuo et al., 2012a). However, until now, a successful allelopathic wheat cultivar has not been obtained. To increase crop resistance to continuous cropping obstacles and autotoxicity and in the selection of crop successions, species’ detoxification potential should be considered as an important indicator of breeding.

## Mechanisms Underlying Allelopathy

Allelopathy has been studied for quite some time, and many aspects of plant physiological and biochemical processes have been proved to be affected by allelochemicals (Zeng et al., 2001; Gniazdowska and Bogatek, 2005). A series of physiological and biochemical changes in plants induced by allelochemicals are detailed as follows.

## Changes in the Micro- and Ultra-Structure of Cells

The shape and structure of plant cells are affected by allelochemicals. Volatile monoterpenes, eucalyptol and camphor can widen and shorten root cells, in addition to inducing nuclear abnormalities and increasing vacuole numbers (Bakkali et al., 2008; Pawlowski et al., 2012). Cruz Ortega et al. (1988) found that a corn pollen extract reduced mitotic activity by more than 50%, induced nuclear irregularities and pyknotic nuclei, and inhibited radicle and hypocotyl growth in watermelon (*Citrullus lanatus* var. *lanatus*). Upon exposure to hordenine and gramine, which are allelochemicals from barley (*Hordeum vulgare*) roots, the radicle tips of white mustard (*Sinapis alba* L.) exhibited damaged cell walls, increases in both the size and number of vacuoles, disorganization of organelles, and cell autophagy (Liu and Lovett, 1993). Likewise, cinnamic acid significantly deformed the ultrastructure of cucumber chloroplasts and mitochondria (Wu et al., 2004). After treatment with benzoic acid, mustard (*Brassica juncea* L.) roots displayed irregularly shaped cells arranged in a disorganized manner and disruption of cell organelles (Kaur et al., 2005). Allelochemicals from *Convolvulus arvensis* L. and catmint (*Nepeta meyeri* Benth.) can alter the random amplification of polymorphic DNA (RAPD) profiles of receiver plants (Kekec et al., 2013; Sunar et al., 2013). Citral is a volatile essential oil component of lemongrass (*Cymbopogon citrates*) and other aromatic plants that has been suggested to have allelopathic traits (Dudai et al., 1999). It was reported that citral can cause disruption of microtubules in wheat and *Arabidopsis thaliana* L. roots, where the mitotic microtubules were more strongly affected than the cortical microtubules (Chaimovitsh et al., 2010, 2012). Moreover, citral has a strong long-term disorganizing effect on the cell ultra-structure of *A. thaliana* seedlings, thickening the cell wall and reducing intercellular communication and the formation of root hairs (Grana et al., 2013).

## Inhibition of Cell Division and Elongation

Allelochemical monoterpenoids (camphor, 1,8-cineole, beta-pinene, alpha-pinene, and camphene) affected cell proliferation and DNA synthesis in plant meristems (Nishida et al., 2005); 2(3H)-benzoxazolinone (BOA) inhibited the mitotic process, especially the G_2_-M checkpoint of lettuce (Sanchez-Moreiras et al., 2008); and sorgoleone reduced the number of cells in each cell division period, damaging tubulins and resulting in polyploid nuclei (Hallak et al., 1999). Burgos et al. (2004) argued that the rye allelochemicals BOA and 2, 4-dihydroxy-1,4(2H)-benzoxazin-3-one (DIBOA) significantly inhibited the regeneration of cucumber root cap cells and thus inhibited growth. Following the treatment of soybean with aqueous leaf extracts from *Datura stramonium* L., Cai and Mu (2012) found that higher concentrations of the extracts inhibited primary root elongation and lateral root development, decreased root hair length and density, inhibited cell division in root tips and increased the chromosomal aberration index and micronucleus index. Teerarak et al. (2012) suggested that the ethyl acetate fraction of *Aglaia odorata* Lour. leaves inhibited mitosis and induced mitotic abnormalities in *Allium cepa* roots by damaging chromatin organization and the mitotic spindle in roots exposed to the allelochemicals.

## Imbalances in the Antioxidant System

The generation and clearing of reactive oxygen species (ROS) and the balance of the redox state in the cell play an important role in allelopathic effects. After exposure to allelochemicals, the recipient plants may rapidly produce ROS in the contact area (Bais et al., 2003; Ding et al., 2007), and alter the activity of antioxidant enzymes such as superoxide dismutase (SOD), peroxidase (POD; Zeng et al., 2001; Yu et al., 2003) and ascorbic acid peroxidase (APX; Zuo et al., 2012b) to resist oxidative stress. Batish et al. (2008) argued that caffeic acid induces significant changes in the activities of proteases, PODs, and polyphenol oxidases (PPOs) during root development and decreases the content of total endogenous phenolics in hypocotyl cuttings from mung bean (*Phaseolus aureus*). Shearer et al. (2012) found that allelopathic interactions led to changes in signal transduction and an imbalance between the production of reactive oxidant species and antioxidant capabilities within a coral holobiont. This oxidative imbalance resulted in rapid protein degradation and ultimately, apoptosis or necrosis of the coral *Acropora millepora* when compensatory transcriptional action by the coral holobiont insufficiently mitigated the damage caused by allelochemicals produced by *Chlorodesmis fastigiata* (Shearer et al., 2012).

## Increases in Cell Membrane Permeability

Many studies have shown that allelochemicals significantly inhibit the activity of antioxidant enzymes and increase free radical levels, resulting in greater membrane lipid peroxidation and membrane potential alteration, which diminish the scavenging effect on activated oxygen and damage the whole membrane system of plants (Lin et al., 2000; Zeng et al., 2001; Lin, 2010; Harun et al., 2014; Sunmonu and Van Staden, 2014). The growth of *Hordeum spontaneum*, *Avena ludoviciana*, and wild mustard seedlings were found to be inhibited by an aqueous extract of barley aerial parts through increasing lipid peroxidation (Farhoudi et al., 2012; Farhoudi and Lee, 2013). Zuo et al. (2012b) argued that the combination of non-sterile shoots of wheat and *Alopecurus aequalis* weeds led to the accumulation of oxygen radical species, such as the superoxide radical O_2_^–^ anion, H_2_O_2_ and malondialdehyde (MDA) in the leaves of transgenic (with Cu/ZnSOD and APX genes) and non-transgenic potato (*Solanum tuberosum* L.) seedlings, in addition to increasing membrane permeability and altering the activities of SOD and APX. Poonpaiboonpipat et al. (2013) found that lemongrass (*Cymbopogon citratus*) essential oil damages the membrane system of barnyard grass (*Echinochloa crus-galli* L.), causing lipid peroxidation and electrolyte leakage. Sun et al. (2014) investigated the generation of ROS induced by pyrogallic acid (PA) in *Microcystis aeruginosa*. They found O_2_^–^ to be the precursor of H_2_O_2_ and showed that the hydroxyl radical OH·was generated at significant levels, demonstrating that PA caused oxidative stress in *M. aeruginosa* and that futile redox cycling of PA was the main source of excessive intracellular O_2_^–^ and consequent H_2_O_2_ and OH·production.

## Effect on the Plant Growth Regulator System

Allelochemicals can alter the contents of plant growth regulators or induce imbalances in various phytohormones, which inhibits the growth and development of plants, for example, with respect to seed germination and seedling growth. Most phenolic allelochemicals can stimulate IAA oxidase activity and inhibit the reaction of POD with IAA, bound GA or IAA to influence endogenous hormone levels (Yang et al., 2005).

Leslie and Romani (1988) found that salicylic acid inhibited the synthesis of ethylene in cell suspension cultures of pear (*Pyrus communis*). Through treatment of wheat seedlings with high concentrations of ferulic acid (2.50 mM), Liu and Hu (2001) found that the growth of wheat seedlings was inhibited by the accumulation of IAA, GA_3_, and CTK, with a simultaneous increase in ABA. An aqueous extract from rice was shown to significantly stimulate IAA oxidase activity in barnyard grass and reduce IAA levels, thereby damaging the growth regulation system and inhibiting seedling growth (Lin et al., 2001). Yang et al. (2008) investigated the mechanisms of two allelochemicals: DTD [4, 7-dimethyl-1-(propan-2-ylidene)-1, 4, 4a, 8a-tetrahydronaphthalene-2, 6(1H, 7H)-dione] and HHO [6-hydroxyl-5-isopropyl-3, 8-dimethyl-4a, 5, 6, 7, 8, 8a-hexahydronaphthalen-2(1H)-one], isolated from *Ageratina adenophora* Sprengel weeds. DTD at a higher concentration (1.5 mM), significantly increased the ABA content in the roots of rice seedlings, but this decreased sharply after 96 h of treatment. HHO also significantly enhanced the ABA content for 48 and 96 h. However, the application of DTD or HHO decreased the IAA and ZR contents in rice roots. The IAA/ABA and ZR/ABA ratios decreased quantitatively in response to higher concentrations of DTO or HHO. These results suggest that the endogenous hormones might have dependent as well as interactive effects on the responses of rice seedlings and their adaptability to DTD or HHO stress. Moreover, the results from another study indicated that cyanamide (1.2 mM) caused an imbalance of plant hormone (ethylene and auxin) homeostasis in tomato (*Solanum lycopersicum* L.) roots (Soltys et al., 2012).

## Effect on the Functions and Activities of Various Enzymes

Allelochemicals exert different effects on the synthesis, functions, contents and activities of various enzymes. Previous studies have shown that the key enzyme λ-phosphorylase involved in seed germination might be inhibited by chlorogenic acid, caffeic acid and catechol (Rice, 1984; Einhellig, 1995). Additionally, POD, CAT, and cellulase can be suppressed by tannic acid, which can also reduce the synthesis of amylase and acid-phosphatase in the endosperm. Phenolic acids can increase the activity of phenylalanine ammonialyase (PAL) and β-glucosidase, while reducing the activity of phenol-β-glucose transferase, thus inhibiting root growth. In addition, protease, invertase and succinodehydrogenase (SDH) can be suppressed by allelochemicals.

Lin et al. (2001) argued that caffeic acid, gallic acid and phenols regulate phenylalanine metabolism by suppressing the activities of PAL and cinnamic acid-4-hydroxylase. An aquatic extract of the above-ground parts and rhizospheric soil of chrysanthemum (*Chrysanthemum indicum* L.) inhibited the activities of root dehydrogenase and nitrate reductase (NiR), reduced the contents of soluble sugar and soluble protein, and inhibited the root growth of stem cuttings of the same species (Zhou et al., 2010). Cheng (2012) investigated the effects of diethyl phthalate (DEP) on the enzyme activity and polypeptide accumulation of glutamine synthetase (GS) in greater duckweed (*Spirodela polyrhiza* L.) and found that DEP is toxic to this species due to the inhibition of GS isoenzymes in nitrogen assimilation and antioxidant enzymes.

## Influence on Respiration

Allelochemicals affect plant growth by influencing different stages of respiration, such as electron transfer in the mitochondria, oxidative phosphorylation, CO_2_ generation and ATP enzyme activity. These chemicals can reduce oxygen intake, which prevents NADH oxidation, inhibits ATP synthesis enzyme activity, reduces ATP formation in mitochondria, disturbs plant oxidative phosphorylation and ultimately inhibits respiration; on the other hand, they can stimulate the release of CO_2_, which promotes respiration.

Cruz Ortega et al. (1988) found that an ethanol extract from corn pollen acted as an inhibitor of the electron pathway and decreased oxygen consumption; the specific inhibition site was most likely located upstream of cytochrome c. Rasmussen et al. (1992) found that sorgoleone interfered with the function of mitochondria isolated from etiolated soybean and corn seedlings by blocking electron transport at the b-c_1_ complex. Moreover, Hejl and Koster (2004b) observed that juglone could reach the mitochondria in the root cells of corn and soybean seedlings, thereby disrupting root oxygen uptake. Alpha-pinene, camphor, limonene and other monoterpenes significantly affect radicle and hypocotyl mitochondrial respiration in soybean and corn, but their targets are different. Alpha-pinene acts under at least two mechanisms: uncoupling of oxidative phosphorylation and inhibition of electron transfer. Alpha-pinene strongly inhibits mitochondrial ATP production, decreases the mitochondrial transmembrane potential and impairs mitochondrial energy metabolism. Camphor causes uncoupling of mitochondria. Limonene inhibits coupled respiration but does not affect basal respiration, and inhibits ATP synthetase and the activities of adenine nucleotide translocase complexes at concentrations of 1.0 and 5.0 mM (Abrahim et al., 2003a,b).

## Effect on Plant Photosynthesis

The impacts of allelochemicals on plant photosynthesis mainly involve inhibition of or damage to the synthesis machinery and acceleration of the decomposition of photosynthetic pigments. Consequently, photosynthetic pigment contents are decreased, which blocks energy and electron transfer, reduces ATP synthesis enzyme activity, inhibits the synthesis of ATP, and affects stomatal conductance and transpiration, which inhibit the photosynthetic process (Meazza et al., 2002; Yu et al., 2003, 2006; Wu et al., 2004). Allelochemicals affect photosynthesis mainly by influencing the function of PS II (Weir et al., 2004; Wang et al., 2014). For example, sorgoleone inhibits the decay of variable fluorescence, blocks the oxidation of the PSII-reduced primary electron acceptor, Q^–^_A_, by the PSII secondary electron acceptor and that of Q_B_ by displacing Q_B_ from the D_1_ protein, thus inhibiting photochemical effects (Gonzalez et al., 1997). Similarly, Shao et al. (2009) demonstrated that the D_1_ protein is an important target in the damage caused to *Microcystis* by pyrogallol. Moreover, Uddin et al. (2012) found that sorgoleone reduced the Fv/Fm of weeds and inhibited weed growth. By studying the inhibitory effect of the dried macroalga *Gracilaria tenuistipitata* (Rhodophyta) on the microalga *Phaeodactylum tricornutum*, Ye et al. (2013) found a decrease in the number of active reaction centers and blockade of the electron transport chain. Poonpaiboonpipat et al. (2013) observed that a high concentration of essential oil from lemongrass (*Cymbopogon citratus*) leaves significantly decreased the chlorophyll a and b and carotenoid contents of barnyard grass and affected alpha-amylase activity in seeds, indicating that essential oil interferes with photosynthetic metabolism. However, aqueous extracts of leaves from *Trema micrantha* (Ulmaceae), an allelopathic plant, did not lead to inhibition of the synthesis of photosynthetic pigments in radish (*Raphanus sativus* L.) (Borella et al., 2014).

## Influence on Water and Nutrient Uptake

Many allelochemicals affect nutrient absorption in plant roots or induce water stress through long-term inhibition of water utilization. Allelochemicals can inhibit the activities of Na^+^/K^+^-ATPase involved in the absorption and transport of ions at the cell plasma membrane, which suppresses the cellular absorption of K^+^, Na^+^, or other ions.

Bergmark et al. (1992) found that ferulic acid (250 μM) inhibited ammonium and NO_3_^–^ uptake in corn seedlings, although ammonium uptake was less sensitive to this treatment than NO_3_^–^. Ferulic acid also inhibits Cl^–^ uptake and increases the initial net K^+^ loss from roots exposed to a low K ammonium nitrate solution and delays recovery that results in a positive net uptake. Yuan et al. (1998) showed that the effects of allelochemicals, such as ferulic acid, benzaldehyde and 4-tert-butylbenzoic acid, on nitrogen absorption in wheat seedlings are negatively correlated, but the negative effects of NH_4_^+^-N on nitrogen absorption were stronger than those of NO_3_^–^-N. Yu and Matsui (1997) observed that cinnamic acid and the root exudates of cucumber inhibited the uptake of NO_3_^–^, SO_4_^2–^, K^+^, Ca^2+^, Mg^2+^, and Fe^2+^ by cucumber seedlings. Through further study, Lv et al. (2002) found that cinnamic acid and p-hydroxybenzoic, the main allelochemicals found in cucumber root exudates, strongly inhibited the activities of root dehydrogenase, root-combined ATPase and nitrate reductase in cucumber, thus inhibiting the root uptake of K^+^, NO_3_^–^, and H_2_PO_4_^–^. Sorgoleone and juglone significantly inhibited H^+^-ATPase activity and the proton-pumping function across the root cell plasmalemma, which affected solute and water uptake in peas (*Pisum sativum* L.), soybeans and corn (Hejl and Koster, 2004a,b). Abenavoli et al. (2010) found that the allelochemicals trans-cinnamic, ferulic acid and p-coumaric acid inhibited net nitrate uptake and plasma membrane H^+^-ATPase activity in maize seedlings, while umbelliferone and caffeic acid had no effect on H^+^-ATPase activity. Sunflower (*Helianthus annus* L.) residues negatively affected plant development, the efficiency of translocation of assimilates and nutrient accumulation in radish plants (Barros de Morais et al., 2014).

The effects of allelochemicals on ion uptake are closely related to allelochemical concentrations and classifications. For example, a low concentration of dibutyl phthalate increases the absorption of N but decreases that of P and K. However, a high concentration of this chemical inhibits the absorption of N, P and K. Similarly, a low concentration of diphenylamine stimulates the absorption of N and K but inhibits the absorption of P by tomato roots (Geng et al., 2009).

## Influence on Protein and Nucleic Acid Synthesis and Metabolism

Most alkaloids show allelopathic potential. Some can closely integrate with DNA and increase the temperature of DNA cleavage, while some can inhibit DNA polymerase I and prevent the transcription and translation of DNA, whereas others can inhibit protein biosynthesis (Wink and Latzbruning, 1995). Allelochemicals can also inhibit amino acid absorption, in addition to transport, thus interfering with protein synthesis, which affects cell growth (Abenavoli et al., 2003). All phenolic acids can affect the integrity of DNA and RNA. Ferulic acid and cinnamic acid as well as many phenols and alkaloids can also inhibit protein synthesis (Baziramakenga et al., 1997; Zeng et al., 2001; Li et al., 2010). This suggests that the observed allelopathic phenomenon is partly a result of the interaction of the allelochemicals with these basic targets, such as DNA, RNA, protein biosynthesis and related processes.

By analyzing the gene expression profile of *A. thaliana* after treatment with fagomine, gallic acid, and rutin, which are allelochemicals found in buckwheat (*Fagopyrum esculentum* Moench), Golisz et al. (2008) observed that genes that reacted to the allelochemicals mainly fell into several functional categories: interaction with the environment, subcellular localization, proteins with a binding function or cofactor requirement, cell rescue, defense and virulence, or metabolism. The plant response to allelochemicals was similar to the response to biotic or abiotic stress. This indicated that allelochemicals might have relevant functions in the cross-talk between biotic and abiotic stress signaling, as they generate ROS (Bais et al., 2003; Baerson et al., 2005; Golisz et al., 2008, 2011). Shao et al. (2009) found that the allelochemical pyrogallol affects the expression of *psbA*, *mcyB*, *prx*, and *faab(* in *Microcystis aeruginosa*, and indicated that membranes are the first target in the damage of *Microcystis* caused by pyrogallol. Guo et al. (2011) showed that HHO affected the expression of *CHS*, which is associated with the synthesis of various amino acids in *Eupatorium adenophorum* roots. Cyanamide alters the expression of the expansin genes, *LeEXPA9* and *LeEXPA18*, which are responsible for cell wall remodeling after cytokinesis, thereby inhibiting the formation of tomato root (Soltys et al., 2012). In a recent study, Fang et al. (2015) found that the expression levels of miRNAs relevant to plant hormone signal transduction, p53 signaling pathways, nucleotide excision repair and the peroxisome proliferator-activated receptor were enhanced in barnyard grass co-cultured with allelopathic rice or treated with rice-produced phenolic acids. Kato-Noguchi et al. (2013) reported that the rice allelochemicals momilactone A and B might inhibit the germination of *Arabidopsis* seeds by inhibiting the degradation process of the storage proteins cruciferin and cruciferina.

Allelochemicals produced by donor plants act on receiver plants, while the receiver plants will react to the donor plants by inducing changes in gene expressions. The up-regulated expression of *PAL*, *cinnamate-4-hydroxylase* (*C4H*), *ferulic acid 5-hydroxylase* (*F5H*), and *caffeic acid O-methyltransferases* (*COMT*), which are involved in the biosynthesis of phenolic compounds in rice, is consistent with their inhibitory effects on barnyard grass, while barnyard grass induces the expression of genes related to the synthesis of phenolic compounds in allelopathic rice (He et al., 2012a).

## Effects of Allelochemicals on Microorganisms and the Ecological Environment

**FIGURE 3:**
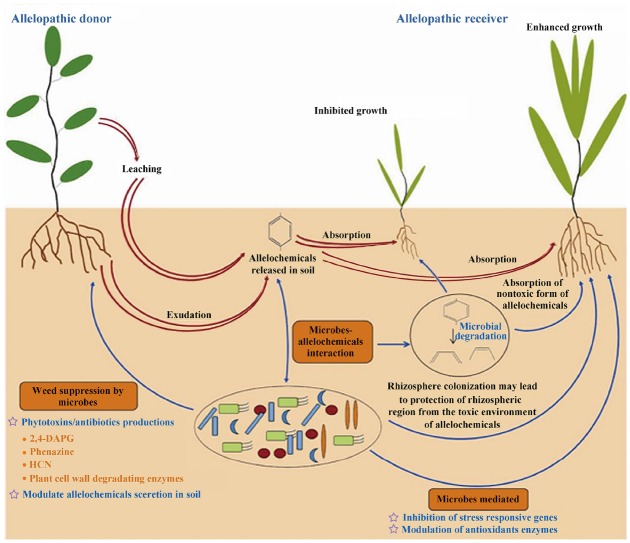
**A schematic diagram showing the various roles of microbes in modulating the interaction of allelopathic donor-receiver species (**Barazani and Friedman, 1999**; **Bais et al., 2006**; **Mishra et al., 2013**).** Red arrows with double lines indicate the phenomenon of allelopathy, and blue arrows with single lines indicate the involvement of various microbial processes in reducing/enhancing allelopathic inhibition by soil microorganisms. This figure explains that beneficial rhizobacteria can minimize the phytotoxicity of the allelopathic donor toward the allelopathic receiver by using various rhizospheric processes such as rhizosphere colonization, biofilm formation, and degradation/transformation of toxic allelochemicals or modulation of the defense mechanism in receiver species by inducing the expression of stress responsive genes or the activity of antioxidant enzymes. Furthermore, microbes also can play an important role in the activation of allelochemicals, e.g., through the release of non-toxic glycosides followed by microbial degradation to release the active allelochemical.

Researchers have found that there are significant relationships between crop growth and soil microbes under the application of allelochemicals or in the presence of allelopathic plants (Figure 3; Barazani and Friedman, 1999; Bais et al., 2006; Mishra et al., 2013). Recent studies demonstrated that indirect effects of allelopathy as a mediator of plant–plant interactions were more important than the direct effects of an inhibitor (Zeng, 2014). Chemical-specific changes in soil microbes could generate negative feedbacks in soil sickness and plant growth (Stinson et al., 2006; Huang et al., 2013; Zhou et al., 2013; Li et al., 2014). Meanwhile, the rhizosphere soil microbes contribute to the allelopathic potential of plants through positive feedback (Inderjit et al., 2011; Zuo et al., 2014; Wu et al., 2015). Bacteria can help to increase inhibition by activating a non-toxic form of an allelochemical (Macias et al., 2003). For example, non-glycosylated compounds may be modified after release from plants and become more toxic (Tanrisever et al., 1987; Macias et al., 2005a). However, bacteria can also help susceptible plants to tolerate biotic stress associated with weeds, and to decrease the allelopathic inhibition of weeds by causing alterations in the expression patterns of some genes that might be responsible for different functions but ultimately lead to a self-defense process (Mishra and Nautiyal, 2012). In addition, the microbial degradation/transformation of allelochemicals in soil affects the effective dose of allelochemicals that can cause plant inhibition (Mishra et al., 2013; Li et al., 2015). Bacterial biofilms in rhizospheric regions can protect colonization sites from phytotoxic allelochemicals and can reduce the toxicity of these chemicals by degrading them (Mishra and Nautiyal, 2012; Mishra et al., 2012). Microorganisms have the ability to alter the components of allelochemicals released into an ecosystem, highlighting their key role in chemical plant–plant interactions and suggesting that allelopathy is likely to shape the vegetation composition and participate in the control of biodiversity in ecology (Fernandez et al., 2013). Some sesquiterpenoid lactones and sulfides are antimicrobial and can disrupt the cell walls of fungi and invasive bacteria, while others can protect plants from environmental stresses that would otherwise cause oxidative damage (Khan et al., 2011; Chadwick et al., 2013). Zhang et al. (2013a) found that antifungal volatiles released from Chinese chive (*Allium Tuberosum* Rottler) helped to control Panama disease (*Fusarium* wilt) in banana (*Musa* spp.) and showed that intercropping/rotation of banana with Chinese chive could control Panama disease and increase cropland biodiversity.

Wang et al. (2013b) indicated that the shift in the microbial community composition induced by barnyard grass infestation might generate a positive feedback in rice growth and reproduction in a given paddy system. The relative abundance and population of plant parasitic nematodes were significantly reduced in the presence of *Chromolaena odorata* (Asteraceae) fallow (Odeyemi et al., 2013). Pearse et al. (2014) found that radish soils had a net positive effect on *Lupinus nanus* biomass and explained that radish might alter the mutualistic/parasitic relationship between *L. nanus* and its rhizobial associates, with a net benefit to *L. nanus*. Fang et al. (2013) indicated that inhibiting the expression of the rice *PAL* gene reduced the allelopathic potential of rice and the diversity of the rhizosphere microflora. These findings suggested that *PAL* functions as a positive regulator of the rice allelopathic potential.

PGPR, such as root-colonizing *Pseudomonas*, *Paenibacillus polymyxa*, endophytes and *Chryseobacterium balustinum* Aur9, have been shown to alter plant gene expression and regulate plant allelochemical synthesis and signaling pathways to enhance disease resistance, adaptability and defense capabilities in response to biotic and abiotic stresses in plants (van Loon, 2007; Dardanelli et al., 2010; Mishra and Nautiyal, 2012).

## Problems and Future Research Directions

Allelochemicals mainly consist of secondary metabolites that are released into the environment through natural pathways, such as volatilization, leaf leaching, residue decomposition, and/or root exudation. Therefore, it should first be noted how allelochemicals are released into the environment (Inderjit and Nilsen, 2003). The activity of allelochemicals varies with research techniques and operational processes (Peng et al., 2004). The natural state of allelochemicals may be changed somewhat during the process of extraction (Li et al., 2002). Therefore, researchers must be careful to determine whether a plant has allelopathic potential or separate and identify allelochemicals using organic solvents and aqueous extracts from plant tissues.

An allelochemical released into the environment is usually not a single substance, and the amounts of allelochemicals released under different conditions vary. Therefore, both the type and amount of allelochemicals released by plants should be considered when their allelopathic potential is investigated. Interactions such as synergy, antagonism and incremental effects between different allelochemicals should be evaluated because one allelochemical may not show allelopathic activity as a single component in a certain situation, but might increase allelopathy in association with other allelochemicals (Albuquerque et al., 2010).

**FIGURE 4:**
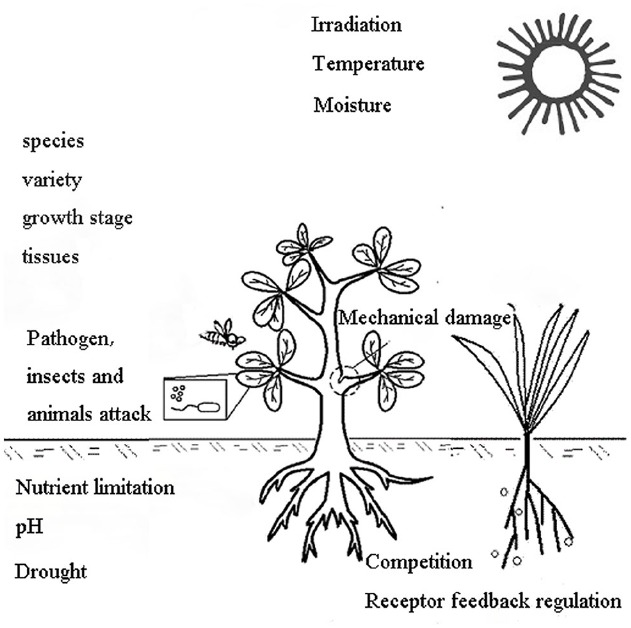
**Induction of allelochemical production by the plant itself and environmental factors (Part of this figure was modified from Albuquerque; **Albuquerque et al., 2010**).** The plant factors include species, variety, growth stage, tissue type, etc. Environmental factors include abiotic factors (irradiation, temperature, nutrient limitation, moisture, pH) and biotic factors (plant competition, diseases, insects, animal attack and receptor feedback regulation).

The type and amount of allelochemicals released into the environment depend on the combined effects of the plant itself (plant factors) and environmental factors, as shown in Figure 4 (Albuquerque et al., 2010). The plant factors include the species, variety, growth stage and different tissues (Belz, 2007; Leao et al., 2012; Iannucci et al., 2013). Allelopathic effects vary between varieties or genotypes (Li and Shen, 2006; Zhou et al., 2011; Leao et al., 2012). Plants from the same environment or with close taxonomic proximity do not necessarily display similar production of secondary metabolites, and they may therefore not secrete the same quantity and quality of allelochemicals or have similar allelopathic effects (Chon and Nelson, 2010; Hagan et al., 2013; Imatomi et al., 2013). Lin et al. (2000) found that varietal differences in the allelopathic potential of rice were related to the genetic background. Environmental factors include both abiotic factors (e.g., irradiation, temperature, nutrient limitation, moisture, pH) and biotic factors (e.g., plant competition, diseases, insects, animal invasion, receptor feedback regulation; Anaya, 1999). In a recent study, endogenous levels of allelochemicals were used as indices of abiotic stress resistance. Meanwhile, the exogenous application of allelochemicals has been found to increase the endogenous level of the receivers, with a simultaneous increase in growth and resistance against abiotic stresses (Maqbool et al., 2013); consequently, appropriate environmental conditions are necessary for allelopathic studies. It has been noted that a stress environment can increase the release of allelochemicals from allelopathic plants (Albuquerque et al., 2010). Through studying the dynamic release of allelochemicals under different stress environments, we can clarify the release characteristics of allelochemicals and determine the conditions required for allelochemical release, thereby revealing the nature of allelochemicals.

Allelochemicals can be degraded after they have been released into the soil; the half-life of allelochemicals varies from a few hours to a few months (Demuner et al., 2005; Macias et al., 2005b; Wang et al., 2007; Barto and Cipollini, 2009; Bertin et al., 2009), and this is mainly associated with the allelochemical concentration, soil type, soil enzymes, and soil microbial population and community structure (Macias et al., 2004; Understrup et al., 2005; Kong et al., 2008; Gu et al., 2009). Previous studies indicated that some allelochemicals had tremendous spatial and temporal heterogeneity (Weidenhamer, 2005; Dayan et al., 2009; Mohney et al., 2009; Weidenhamer et al., 2009, 2014), but these characteristics of most allelochemicals have not been confirmed. It was reported that polydimethylsiloxane (PDMS) microtubing (silicone tubing microextraction, or STME) could be used as a tool to provide a more finely resolved picture of allelochemical dynamics in the root zone (Weidenhamer, 2005; Mohney et al., 2009; Weidenhamer et al., 2009, 2014). Until now, much remains unknown about the fate or persistence of allelochemicals in the soil or their effects on soil chemistry or microflora (Belz, 2007).

Explaining how allelochemicals function is complicated due to the many classes of chemicals and different structures that have been identified as agents in allelopathy. There is no generic allelochemical, and we should certainly anticipate different mechanisms of action among allelopathic chemicals. Moreover, it should be investigated in future studies whether allelochemicals are absorbed through transport proteins or whether different allelochemicals have the same molecular targets in different species (Weston and Mathesius, 2013). The systematic study of allelochemical detoxification mechanisms in different species will help reveal the differences in detoxification mechanisms between plants and microbes.

Allelopathy is a complex process. Many allelochemicals have been identified to date. Due to the different sensitivities of different receptors to the same allelochemical and the various allelopathic activities of different allelochemicals, considerable further work is required in the field of allelochemical research. Very little is known about the transportation and biodegradation of allelochemicals in soil or the population genetics of allelopathic species, the establishment of practical ways of using allelochemicals in the field, the rapid adaptation of weeds to avoid them, the diversity of the soil microbial community that is maintained in their presence or the role of signal transduction in herbivore defense. These areas should be the focus of future investigations.

Considerable research has showed that allelopathy has good application potential in agricultural production. Until now, many allelopathic crops have been used in agricultural production, but the applications are limited to small-scale and regional areas. The structure and mode of action of many allelochemicals have been deeply revealed in recent years, and this has laid a good foundation for projects where allelochemicals are used to obtain the basic structures or templates for developing new synthetic herbicides.

The commonly used methods of weed control (herbicide application, mechanical weeding and hand weeding) are effective in agricultural production. However, there are many disadvantages associated with these methods, for example, the evolution of herbicide resistance in weeds, the negative impacts of herbicides on environmental, human and animal health, the expense of herbicides, the losses in soil structure and the enormous labor requirements. Many of the above problems can be allayed by creating diversity in weed control practices with the application of allelopathy. The combination of more than one weed control method has been proved to be effective in reducing the probability of herbicide resistance development in weeds. Moreover, the combined application of reduced synthetic herbicides dose and allelopathic extracts can provide control that is as effective as that obtained from the standard dose of herbicides (Farooq et al., 2011). Further, using diverse weed management practices in certain fields can ensure sustainable and effective weed control.

## Conclusion

Allelopathy has been known and used in agriculture since ancient times; however, its recognition and use in modern agriculture are very limited. Allelopathy plays an important role in investigations of appropriate farming systems as well as in the control of weeds, diseases and insects, the alleviation of continuous cropping obstacles, and allelopathic cultivar breeding. Furthermore, allelochemicals can act as environmentally friendly herbicides, fungicides, insecticides and plant growth regulators, and can have great value in sustainable agriculture. Although allelochemicals used as environmentally friendly herbicides has been tried for decades, there are very few natural herbicides on the market that are derived from an allelochemical. However, there are a few research investigations testing natural-product herbicides. With increasing emphasis on organic agriculture and environmental protection, increasing attention has been paid to allelopathy research, and the physiological and ecological mechanisms of allelopathy are gradually being elucidated. Moreover, progress has been made in research on the associated molecular mechanisms. It is obvious that allelopathy requires further research for widespread application in agricultural production worldwide.

### Conflict of Interest Statement

The authors declare that the research was conducted in the absence of any commercial or financial relationships that could be construed as a potential conflict of interest.

## Abbreviations

APX: ascorbic acid peroxidase
BNI: biological nitrification inhibition
BNIS: biological nitrification inhibition substances
BOA: 2(3H)-benzoxazolinone
*C4H*: *cinnamate-4-hydroxylase*
CAT: catalase
*COMT*: *caffeic acid O-methyltransferases*
DEP: diethyl phthalate
DIBOA: 4-dihydroxy-1,4(2H)-benzoxazin-3-one
DTD: [4, 7-dimethyl-1-(propan-2-ylidene)-1, 4, 4a, 8a-tetrahydronaphthalene-2, 6(1H, 7H)-dione]
*F5H*: *ferulic acid 5-hydroxylase*
GR: glutathione reductase
GS: glutamine synthetase
HHO: [6-hydroxyl-5-isopropyl-3, 8-dimethyl-4a, 5, 6, 7, 8, 8a-hexahydronaphthalen-2(1H)-one]
ISR: induced systemic resistance
MDA: malondialdehyde
NiR: nitrate reductase
NIS: nitrification-inhibiting substances
PA: pyrogallic acid
PAL: phenylalanine ammonialyase
PDMS: polydimethylsiloxane
PGPR: plant growth-promoting rhizobacteria
POD: peroxidase
PPO: polyphenol oxidase
QTL: quantitative trait locus
RAPD: random amplification of polymorphic DNA
ROS: reactive oxygen species
SDH: succinodehydrogenase
SOD: superoxide dismutase
STEM: silicone tubing microextraction.
