# Corrigendum: Research Progress on the use of Plant Allelopathy in Agriculture and the Physiological and Ecological Mechanisms of Allelopathy

**DOI:** 10.3389/fpls.2016.01697

**Published:** 2016-11-08

**Authors:** Fang Cheng, Zhihui Cheng

**Affiliations:** Department of Vegetable Science, College of Horticulture, Northwest A&F UniversityYangling, China

**Keywords:** allelochemical, allelopathy, agriculture practice, physiological mechanism, ecological mechanism, microorganism, agricultural sustainable development

There was a mistake in Figure 1 Structures of some of the allelochemicals produced by plants. The structural formula of ferulic acid appeared twice. The repeated structural formula has been replaced by another plant allelochemical coumarin. The correct version of Figure 1 appears below. The authors apologize for the mistake. This error does not change the article in any way.

**Figure 1:**
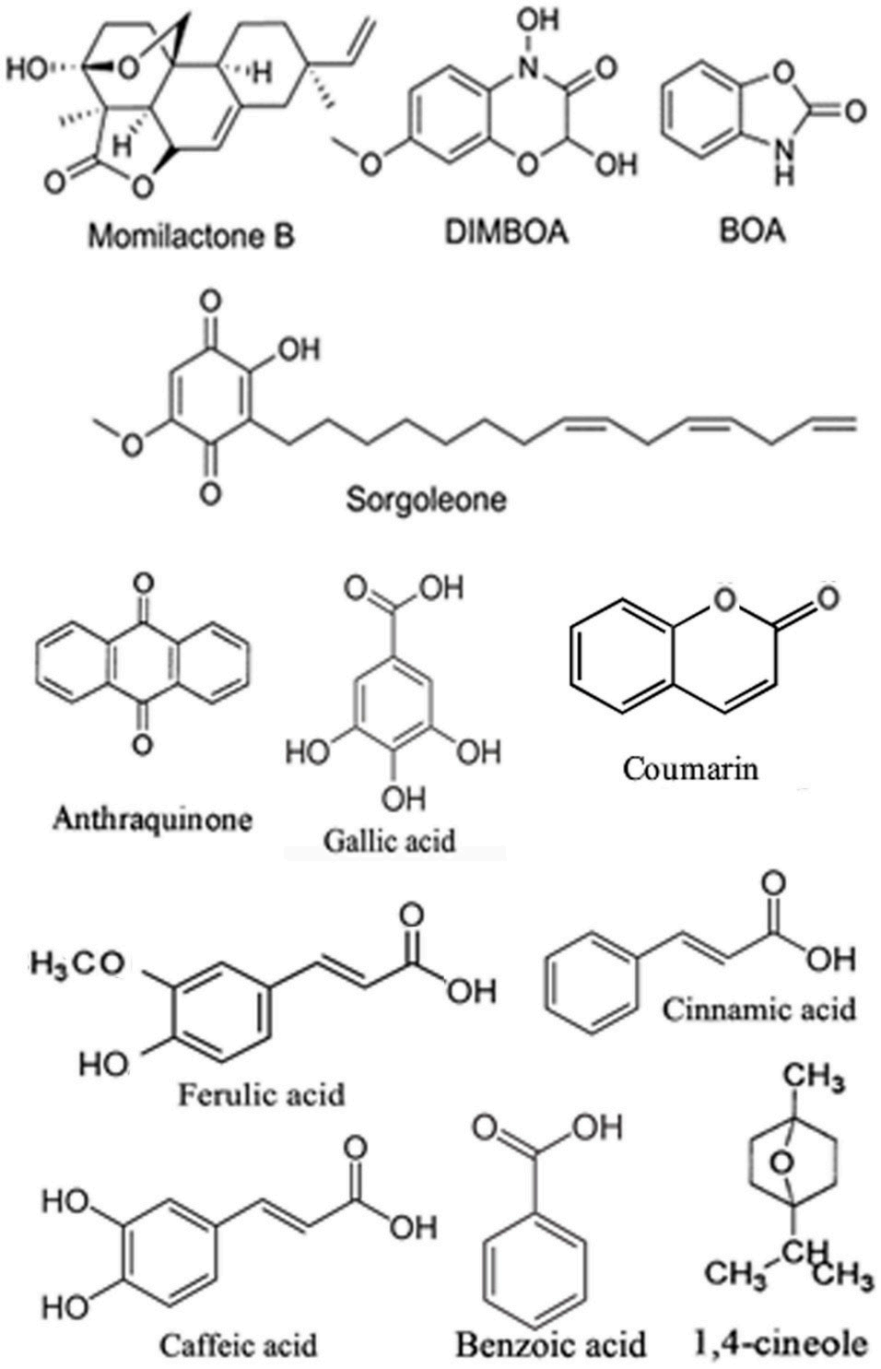
**Structures of some of the allelochemicals produced by plants**.

## Author contributions

All authors listed, have made substantial, direct and intellectual contribution to the work, and approved it for publication.

## Funding

This research and the writing of this review were supported by a project of the National Natural Science Foundation of China (No. 31471865).

### Conflict of interest statement

The authors declare that the research was conducted in the absence of any commercial or financial relationships that could be construed as a potential conflict of interest.

